# Administration of Inulin-Supplemented Gluten-Free Diet Modified Calcium Absorption and Caecal Microbiota in Rats in a Calcium-Dependent Manner

**DOI:** 10.3390/nu9070702

**Published:** 2017-07-06

**Authors:** Urszula Krupa-Kozak, Lidia H. Markiewicz, Grzegorz Lamparski, Jerzy Juśkiewicz

**Affiliations:** 1 Department of Chemistry and Biodynamics of Food, Institute of Animal Reproduction and Food Research of the Polish Academy of Sciences, Tuwima St., 10, 10-748 Olsztyn, Poland; 2 Department of Immunology and Food Microbiology, Institute of Animal Reproduction and Food Research, Polish Academy of Sciences, Tuwima St., 10, 10-748 Olsztyn, Poland; l.markiewicz@pan.olsztyn.pl; 3 Sensory Laboratory, Institute of Animal Reproduction and Food Research, Polish Academy of Sciences, Tuwima St., 10, 10-748 Olsztyn, Poland; g.lamparski@pan.olsztyn.pl; 4 Department of Biological Function of Food, Institute of Animal Reproduction and Food Research, Polish Academy of Sciences, Tuwima St., 10, 10-748 Olsztyn, Poland; j.jaskiewicz@pan.olsztyn.pl

**Keywords:** prebiotics, gluten-free bread, intestinal microbiota, SCFA, calcium balance

## Abstract

In coeliac disease (CD), the risk of adverse calcium balance and reduced bone density is induced mainly by the disease, but also by a gluten-free diet (GFD), the only accepted CD therapy. Prebiotics through the beneficial impact on intestinal microbiota may stimulate calcium (Ca) absorption. In the present study, we hypothesised that the dietary inulin in GFD would influence positively the intestinal microbiota, and by that will stimulate the absorption of calcium (Ca), especially in the conditions of Ca malnutrition. In a six-weeks nutritional experiment on growing a significant (*p* < 0.05) luminal acidification, decrease in ammonia concentration and stimulation of short chain fatty acids formation indicated inulin-mediated beneficial effects on the caecal microbiota. However, the effect of inulin on characteristics of intestinal microbiota and mineral utilization depended on the dietary Ca intake from GFDs. Inulin stimulated bifidobacteria, in particular *B. animalis* species, only if a recommended amount of Ca was provided. Most benefits to mineral utilization from inulin consumption were seen in rats fed Ca-restricted GFD where it increased the relative Ca absorption. Administration of inulin to a GFDs could be a promising dietary strategy for beneficial modulation of intestinal ecosystem and by that for the improvement the Ca absorption.

## 1. Introduction

Calcium (Ca) is an important macroelement of the human body and in majority it is deposited in bones providing the structural integrity of the skeleton. Ca homeostasis is precisely controlled with coordinated action of processes such as absorption in the intestine, reabsorption from the kidney and exchange from bones. The intestinal Ca absorption is essential to ensure the appropriate concentrations of intra- and extracellular calcium fluids without bone depletion [1]. On the other side, bones have a metabolic function since Ca is continuously exchanged between bone and blood, and can be released from bone to maintain extracellular calcium concentrations, regardless of intake. 

Calcium requirements vary throughout an individual’s life, with greater needs during the periods of rapid growth in childhood and adolescence [2]. Bone density increases until the end of puberty, when it reaches its peak value. If a normal peak bone mass is not achieved, the individual is at a higher risk for developing osteoporosis; thus, the amount of bone accrued during the paediatric years is an important predictor of an individual’s future resistance to fractures [3]. Nevertheless, even an adequate dietary Ca intake may not ensure a proper calcium balance. Besides the amount, its absorption is a critical factor determining Ca bioavailability for bone development and maintenance. In some cases, an adverse calcium balance is observed that may result from a poor intestine absorption caused by infection, inflammation or pathology in the intestine morphology. Among the group of the calcium deficiency risk there are individuals suffering from chronic intestinal diseases, including coeliac disease (CD). 

In CD, defined as a permanent gluten (mixture of proteins found in wheat, rye, and barley) intolerance with genetic etiology [4], a chronic intestinal inflammation and malabsorption of calcium and vitamin D are observed that together with general malnutrition affects negatively bone health [5]. Recently, a persistent villus atrophy has been associated with serious sequelae, including osteoporotic fractures [6]. Several studies have demonstrated a low bone mineral density (BMD) both in children and adults with CD [7,8]. Additionally, a risk for less-than-optimal peak bone mass acquisition and a retarded growth in CD children is observed. These adverse alterations are mainly induced by the underlying disease, but, to a certain degree, also by a gluten-free diet (GFD) [9,10], which is the only accepted therapy for CD [11]. Above 80% of CD children adhering to a GFD consume lower than recommended amounts of Ca [12,13] and vitamin D, which could be due to the reduced nutritional quality of a GFD [14,15]. 

Nutrition plays an important role in proper bones mineralization [16]. To reduce a risk of osteoporosis, an increased calcium intake is proposed as one of the most effective strategy. However, when Ca intake remains inadequate, an improvement of Ca absorption becomes an important method of Ca balance restoration and bone health improvement. Among the dietary compounds, prebiotics are widely studied functional ingredients which can enhance the mineral absorption and bone properties [17,18]. Inulin, a polydisperse carbohydrate material consisting mainly of beta (2-1) fructosyl-fructose links, is an example of prebiotic naturally occurring in tubers, bulbs and tuberous roots of several edible fruits and vegetables [19]. Inulin and other inulin-type fructans (ITFs) are resistant to digestion in the small intestine and undergo fermentation in the large intestine, resulting in short-chain fatty acid (SCFA) production. On the other hand, they stimulate growth and/or activity of selected commensal bacteria, including the health-promoting bifidobacteria and lactobacilli [20]. Several animal studies have shown that the acidification of the gut environment resulting from bacteria fermentation of ITFs enhanced calcium and magnesium absorption and bone mineralization [21,22,23] nevertheless, the human studies have shown contentious results [24,25,26]. In the present study, we hypothesised that the dietary administration of inulin would influence positively the intestinal microbiota, and by that will stimulate the absorption of Ca from colon and caecum of rats, especially in the conditions of Ca malnutrition. To verify this hypothesis, a 6-weeks nutritional experiment was performed aimed to assess the impact of dietary inulin on gastrointestinal tract parameters, characteristics and activity of gut microbiota (SCFAs, PSCFAs, microbial enzymes), and mineral utilization in growing rats fed GFD with reduced Ca content, established as an experimental model that in some extend may correspond to a dietary conditions observed in many paediatric CD patients treated with GFD.

## 2. Materials and Methods

### 2.1. Composition of Experimental Gluten-Free Diets

The experimental GFDs were composed mainly of gluten-free breads with Ca content recommended for rats (O) or gluten-free breads with reduced (R) Ca content, supplemented or not supplemented with inulin (Frutafit HD, Hortimex, Konin, Poland) (Table 1). Gluten-free breads were baked in the laboratory conditions [27], dried (at RT/for 24 h) and ground into a powder. The only source of dietary Ca were Ca salts applied in gluten-free breads, in particular calcium caseinate (Lacpol Company, Murowana Goślina, Poland) and calcium citrate (Hortimex, Konin, Poland). All experimental GFDs were additionally supplemented with dl-Methionine, soybean oil (“Kruszwica” SA Company, Kruszwica, Poland), a Ca-free mineral mix and a vitamin mix (Table 1). The energy density of each GFD was calculated by multiplying the amount of each macronutrient (Table S1) by the corresponding conversion factor according to FAO recommendations [28].

### 2.2. Animals

The study was conducted on 32 male growing Wistar rats (aged 4 weeks) with similar initial body weight (103 ± 4 g). All experimental protocols applied in the study were approved by the Institutional Laboratory Animal Care and Use Committee (Olsztyn, Poland, Permit Number: 15/2007/N). The experiment was conducted in compliance with European guidelines for the care and use of laboratory animals.

### 2.3. Experimental Design

Our approach was conducted in the growing rats model that in some extend may resemble children who are characterised by a greater Ca need to achieve the peak bone mass and bone strength during growth. The experimental model of dietary Ca shortage was established to mimic the conditions of insufficient dietary Ca intake that may occur in paediatric CD patients treated with GFD. Rats were divided into four experimental groups (8 per group): O-fed GFD with the recommended Ca content; OI-fed GFD with the recommended Ca content and inulin; R-fed GFD with reduced Ca content; and RI-fed GFD with reduced Ca content and inulin. The animals were housed individually in metabolic plastic cages under standard conditions at a temperature of 21–22 °C and a relative air humidity of 50–70% with intensive room ventilation and a 12-h lighting regiment. For 6 weeks, rats of each group were fed *ad libitum* one of the four experimental diets, with continuous access to distilled water.

### 2.4. Analysis of Food Intake, Body Weight Gain and Minerals Bioavailability

The food intake and body weight gains of individual rats were recorded daily throughout the study. The bioavailability of Ca, Mg and P, denoted by coefficients of apparent absorption and retention, was analysed in a nutritional experiment. After the preliminary period (10 days), faeces and urine were collected daily for 5 consecutive days from all rats kept in balance cages (Tecniplast Spa, Buguggiate, Italy). The content of Ca, Mg and P in diets, as well as in faeces and urine collected in the balance period was determined by atomic absorption spectroscopy (AAS). The coefficient of apparent absorption was calculated as the difference between the intake of minerals and their quantity excreted with faeces, and it was expressed in relative (%) values. The coefficient of apparent retention was calculated as the difference between the intake of minerals and their quantity excreted with faeces and urine, and expressed in relative (%) values.

### 2.5. Sampling Procedures

At the end of the experiment, rats were anaesthetised with sodium pentobarbitone according to euthanasia guidelines for experimental animals (50 mg/kg body weight) [30]. Blood samples were collected from the tail vein, placed in plastic tubes and left for 1 h at room temperature to aggregate erythrocytes. After laparotomy, the small intestine, caecum and colon with the contents were collected and weighed. The pH of the small intestinal, caecal and colonic digesta was measured immediately (ca. 10 min), directly in segments (model 301 pH meter; Hanna Instruments, Vila do Conde, Portugal). Samples of fresh caecal digesta were used for an immediate determination of ammonia content, dry mass, bacterial enzyme activity and SCFA content, while the rest of the caecal digesta was transferred to tubes and stored at −70 °C for the molecular characteristics of microbiota. Duodenal, caecal and colonic walls were flushed clean with ice-cold physiological saline, blotted on filter paper and weighed for tissue mass. The blood was centrifuged for 15 min at 2500× *g* and 4 °C, and the obtained serum was then stored at −70 °C until minerals analysis.

### 2.6. Analytical Procedures

#### 2.6.1. Ammonia and Dry Matter (DM) Content

The content of ammonia, which was extracted from the caecal digesta and trapped in a solution of boric acid, was quantified by direct titration with sulphuric acid acc. to Hofirek & Haas method [31]. The dry matter (DM) content of caecal digesta was determined at 105 °C.

#### 2.6.2. Short Chain Fatty Acids

The concentrations of SCFAs in samples of caecal digesta samples were determined by gas chromatography (Shimadzu GC-14A, Kyoto, Japan) according to a previously described method [32]. Briefly, caecal digesta samples (0.2 g) were mixed with 0.2 mL formic acid, diluted with deionised water and centrifuged at 7211× *g* for 10 min. The supernatant was loaded onto a 2.5 m × 2.6 mm glass column, containing 10% SP–1200/1% H_3_PO_4_ on 80/100 Chromosorb W AW (Supelco, Bellefonte, PA, USA). Column temperature was 110 °C; the temperature of the flame ionisation detector (FID) was 180 °C, and the injection temperature was 195 °C. The caecal SCFA pool was calculated as the concentration of SCFA in the caecum (μmol/g) multiplied by the weight of caecal contents (g), and it was expressed in μmol per 100 g of body mass. The concentrations of caecal putrefactive SCFAs (PSCFAs) were calculated as the total content of isobutyric, isovaleric and valeric acid. All SCFAs analyses were performed in duplicate.

#### 2.6.3. Activity of Bacterial Enzymes

The activity of selected bacterial enzymes in the caecal digesta was measured based on the rates of *p*- and *o*-nitrophenol release from their nitrophenyl glucosides according to a previously described method [32]. The activity of α- and β-glucosidases, α- and β-galactosidases, and β-glucuronidase was expressed in μmol of the product synthesised per min (unit) per gram of digesta in a fresh caecal sample.

#### 2.6.4. Microbiota Characteristics with PCR-DGGE

DNA was extracted from ca. 0.1 g of the caecal digesta using the GeneMATRIX Bacterial and Yeast Genomic DNA Purification kit (Eurx, Gdańsk, Poland) and the bead-beating method according to the manufacturer’s protocol. Variable regions of 16S rRNA gene were amplified with the use of universal, group- and genus-specific primer sets (Table S2), and were subsequently separated in polyacrylamide gels with a denaturing gradient of formamide and urea (Table S3) as described by Markiewicz et al. [33]. The profiles of total bacteria (TB), *Lactobacillus* (LAC), *Bacteroides* (BPP), and the *C. leptum* group (Clept) were determined. Amplifications were carried out in the C1000 thermal cycler (Bio-Rad, Warsaw, Poland) in a total volume of 30 µL comprising of a reaction buffer (3 μL), of JumpStart Taq DNA polymerase (1.25 U, Sigma, Poznań, Poland), MgCl_2_ (Table S1), dNTP (200 μM), the template DNA (2 μL), filled up with sterile deionized water to 30 μL. The reaction program included: one cycle at 95 °C for 5 min, 35 cycles at 95 °C for 20 s, annealing temperature (Table S1) for 20 s, 72 °C for 20 s and the final cycle at 72 °C for 20 min. The PCR product (20 μL) was separated in 8%, polyacrylamide gel (acrylamide/bisacrylamide 37.5:1) in the 0.5× TAE buffer at 60 °C. Denaturing gradients and electrophoresis conditions are shown in Table S2. Gels were stained with SybrGreen I dye (Sigma) in the 1× TAE buffer according to the supplier’s recommendations and photographed under UV light (Gel Doc™ XR, Bio-Rad). Selected DGGE bands were cut out from a gel with a sterile scalpel and incubated overnight in 40 μL of the TE buffer at 4 °C. Two microlitres of the aliquots were used as a template in the re-amplification reactions which were conducted under the previously described conditions. The re-amplified DNA was purified using the GeneMATRIX PCR/DNA Clean-Up Purification Kit (Eurx). The DNA was commercially sequenced by Genomed (Warsaw, Poland). The obtained sequences were identified using a BLASTn tool. 

#### 2.6.5. Quantification of Caecal Microbiota by Real-Time PCR

Real-time PCR method with the use of universal and group- and genus-specific primers was performed according to the procedure described by Fotschki et al. [34]. Briefly, a reference standard containing DNA isolated from known number of intestinal bacteria cells was prepared. DNA was isolated by the method described in above (point 2.6.4) from a total of 3.33 × 10^9^ bacterial cells consisting of 3.18 × 10^8^ cells of *Bacteroides*, 3.99 × 10^7^ of the *Clostridium leptum* group (clostridial cluster IV), 1.44 × 10^9^ of *Bifidobacterium*, 1 × 10^9^ of *Enterococcus*, and 5.35 × 10^8^ of *Lactobacillus* cells. Decimal dilutions of the DNA standard were used to plot a standard curve with each primer pair used (Table S4). Amplifications were performed in the iQ5 real-time PCR system (Bio-Rad) in a total volume of 25 μL (12.5 μL of SYBR Green Jump-Start Taq ReadyMix (Sigma), 1 μL of 10-fold diluted DNA, 200 μM of each primer, and PCR-grade water). The temperature program included 1 cycle of 95 °C for 3 min and 35 cycles of 95 °C for 20 s, primer annealing temperature (Table S4) for 30 s, and 72 °C for 30 s with signal acquisition. After each run a melting curve was prepared to confirm the specificity of amplicons. Samples were run in duplicate. The obtained values were normalized according to the dilution and weight of the sample. The results were expressed as log10 of the number of cells per gram of wet weight of a sample.

#### 2.6.6. Mineral Concentration

Mineral concentrations in the biological materials were measured by flame (air—acetylene burner) atomic absorption spectrometry method (AAS) using an atomic absorption spectrophotometer (iCE 3000 SERIES, Thermo Fisher Scientific Inc., Waltham, MA, USA) equipped with an autosampler and the appropriate cathode lamp operating at the resonance line of the analysed bioelements (Ca: 422.7 nm; Mg: 285.2 nm). Before analysis, the samples were wet-digested with a mixture (9:1; *v*/*v*) of concentrated nitric acid (65% HNO_3_; Merck, Darmstadt, Germany) and hydrochloric acid (30% HCl; Merck) using a microwave system (Multiwave, Anton Paar GmbH, Graz, Austria). Calcium concentration was validated by adding a solution of lanthanum (III) chloride hydrate (LaCl_3_ × 7H_2_O; Merck, Germany) to all samples in sufficient amounts to obtain 0.5% concentration of La^3+^. Phosphorus content was determined by the colorimetric molybdate method with hydroquinone (POCH S. A., Gliwice, Poland) and sodium (IV) sulphate (POCH S. A., Gliwice, Poland). Absorbance was measured using the VIS 6000 Spectrophotometer (KRÜSS–OPTRONIC, Hamburg, Germany) at *λ* = 610 nm. The concentrations of Ca, Mg and P were automatically read from a calibration curve (Ca: range 0.5–4.0 µg/mL; Mg: range 0.05–0.8 µg/mL; P: range 0.4–2.0 µg/mL) prepared with the AAS standard solution of Ca, Mg and P, respectively (J.T.Baker^®^ Chemicals, Avantor, Center Valley, PA, USA) and expressed as mM/L or mg/g. The analyses were repeated (*N* = 8) for analytical quality control. 

### 2.7. Statistic Analysis

The physiological responses of the treated animals were expressed by a mean of 8 values with standard deviation (±SD). The calculations were performed in STATISTICA 6.0 (StatSoft Corp., Kraków, Poland) software. Two-way ANOVA was performed to assess the effect of Ca level (recommended or reduced; Ca), the effects of dietary inulin (diets with and without inulin; I), and the interactions between the investigated factors (Ca × I). When significant treatment effects were found in the ANOVA, the post-hoc comparisons were performed using the Duncan’s multiple range test. The data were checked for normality before statistical analyses. Differences *p* < 0.05 were considered significant. 

The frequency of bacterial taxa (*Olsenella*) in the microbiota was compared with the Fisher’s exact test performed in STATISTICA 6.0 (StatSoft Corp.). Differences were regarded as statistically significant at *p* < 0.05. DGGE banding profiles were processed using the BioNumerics software (Applied Maths, Sint-Martens-Latem, Belgium). Gels were normalized to one sample (as an external standard) run for each gel set. Profile similarities were calculated using the Pearson’s product-moment correlation coefficient, and dendrograms were constructed using the Unweighted Pair Group Method with Arithmetic Mean (UPGMA) algorithm. The similarity matrices obtained during analyses of DGGE patterns of eubacteria, *Bacteroides*, *C. leptum* group and *Lactobacillus* were used for composite data set analysis to calculate average profile similarities. The generated similarity matrix was used to develop a multidimensional scaling (MDS) diagram [35].

## 3. Results

### 3.1. Effect of a Gluten-Free Diet Enriched with Inulin on a Daily Food Intake, Body Weight Gain and Gastrointestinal Tract Parameters

The intake of dietary Ca (recommended or restricted) and the addition of inulin to experimental GF diets had no significant effect (*p* > 0.05) on the daily food intake or body weight gains (BWG) of animals during the 6 weeks study (Table 2). However, these dietary factors influenced the majority of intestinal parameters, both alone and in combination. A two-way ANOVA revealed that independently of dietary Ca intake, the experimental GFDs containing inulin significantly increased the relative weight of the small intestine and the acidity of digesta in this segment of the gastrointestinal tract (*p* < 0.05 in both cases). The interaction between dietary Ca intake and inulin significantly influenced the weight of the examined tissues and accumulated digesta in both analysed segments of the large intestine (*p* < 0.001 for the caecum and *p* < 0.05 for the colon, respectively). The DM content of caecal digesta and the concentration of caecal ammonia were inulin-dependent, and a significant reduction in DM content and ammonia concentration (*p* = 0.0346 and *p* < 0.001, respectively) was noted in groups fed a GFD containing inulin (Table 2). The significant acidification of the caecal and colonic environment was attributed to both Ca intake (*p* = 0.0019 and *p* < 0.001, respectively) and inulin intake (*p* < 0.001 in both cases).

### 3.2. Effect of a Gluten-Free Diet Enriched with Inulin on the Concentration of Short Chain Fatty Acids (SCFAs), Putrefactive SCFAs (PSCFAs) and Their Profiles in the Caecal Digesta

The results of two-way ANOVA revealed that the total concentration of SCFAs in the caecal digesta was significantly (*p* < 0.01) affected by the interaction between both dietary variables, Ca intake and inulin intake (Table 3). These resulted mainly from changes in the concentration of straight SCFAs. The experimental GFD with the recommended Ca content and inulin stimulated the formation of propionate and butyrate, whereas the opposite effect was observed when Ca intake was limited. The concentration of putrefactive SCFAs (PSCFAs) in the caecal digesta, determined as the sum of *iso*-butyric, *iso*-valeric and valeric acids, was significantly (*p* < 0.05) reduced by restricted intake of dietary Ca and administration of inulin. The interaction between Ca and inulin intake significantly affected (*p* < 0.05) the profiles of three major SCFAs (Table 3). Both insufficient intake of dietary Ca and inulin supplementation significantly reduced the ratio of acetic acid to total SCFAs, but increased the ratio of propionate to total SCFAs. 

### 3.3. Effect of a Gluten-Free Diet Enriched with Inulin on the Activity of Bacterial Enzymes in the Caecal Digesta

The activity of bacterial enzymes α- and β-glucosidase, α- and β-galactosidase, and β-glucuronidase in the caecum was analysed at the end of the 6-weeks study (Table 4). The results of a two-way ANOVA indicate that analysed variables, a dietary calcium intake and inulin intake stimulated significantly the activity of α-glucosidase and β-galactosidase in an independent manner (no interaction). While, their interaction (Ca × I) had a significant (*p* < 0.001) effect on activity of β-glucosidase and β-glucuronidase. The activity of bacterial β-glucuronidase was effectively reduced by lower dietary Ca intake as well as by inulin in the experimental diets (*p* < 0.001 for both variables).

### 3.4. Effect of a Gluten-Free Diet Enriched with Inulin on the Quantitative Profile of Caecal Microbiota

**Figure 1:**
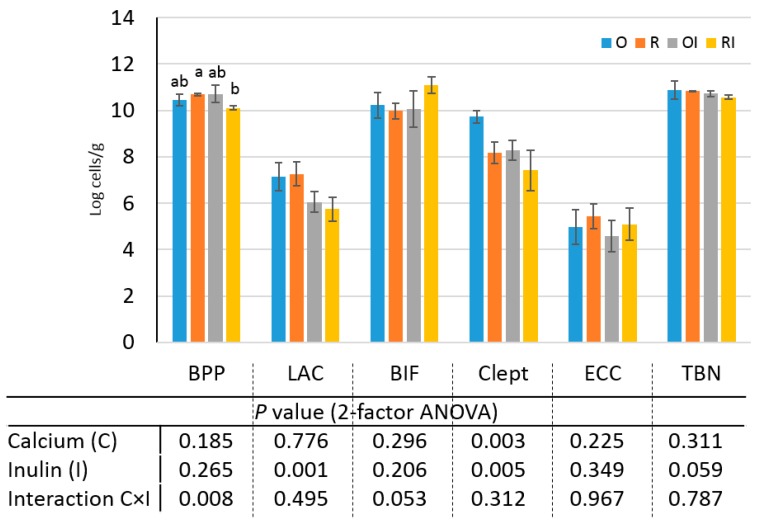
Counts of *Bacteroides-Prevotella-Porphyromonas* (BPP), *Lactobacillus* (LAC), *Bifidobacterium* (BIF), *Clostridium leptum* group (Clept), *Enterococcus* (ECC) and total bacterial number (TBN) of caecal microbiota of rats fed the experimental GFDs. Values are expressed as means ± standard deviation; O, group fed a GF diet with the recommended calcium content; R, group fed a GF diet with restricted calcium content; OI, group fed a GF diet with the recommended calcium content and inulin; RI, group fed a GF diet with restricted calcium content and inulin. Significant differences between groups O, R, OI and RI are indicated with superscripts only when C × I interactions were statistically significant (*p* < 0.05).

An analysis of the quantitative profile of caecal microbiota revealed that neither restricted Ca intake nor inulin supplementation influenced the total bacteria number (TBN) and *Enterococcus* (Figure 1). Inulin administration affected *Lactobacillus* counts (*p* = 0.001) independently of dietary Ca intake, whereas both restricted Ca intake and inulin intake lowered the counts of bacteria of *Clostridium leptum* group (Figure 1) in an independent manner (no interaction). The *Bifidobacterium* count tended (*p* = 0.053) to be increased by Ca × I interaction, whereas *Bacteroides-Prevotella-Porphyromonas* counts were significantly affected by an interaction between the two investigated factors (*p* < 0.01).

### 3.5. Effect of a Gluten-Free Diet Enriched with Inulin on the Qualitative Profile of Caecal Microbiota

**Figure 2:**
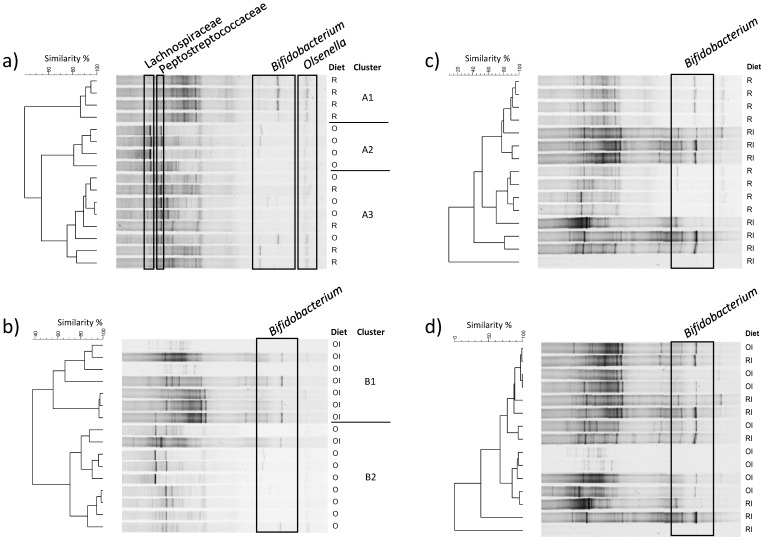
DGGE banding patterns of caecal bacteria obtained with universal primers. Frames depict gel positions assigned to a specific bacterial taxon based on band sequencing (see Supplementary Information, Figure S1). O and R—diets with recommended and restricted calcium intake, respectively. OI and RI—diets O and R supplemented with inulin. Paired comparisons, O vs. R (**a**), O vs. OI (**b**), R vs. RI (**c**) and OI vs. RI (**d**), were performed based on profile similarities calculated using the Pearson’s product-moment correlation coefficient. Dendrograms were constructed using the UPGMA algorithm.

A qualitative analysis (PCR-DGGE) of bacterial groups influenced by the experimental GFDs was performed based on the results of a quantitative analysis of caecal microbiota. Based on the sequenced DGGE bands, gel regions were assigned to a specific bacterial family (*Lachnospiraceae*, *Peptostreptococcaceae*, *Erysipelotrichaceae* and *Selenomonadaceae*) or genus (*Bifidobacterium*, *Olsenella*) (Figure S1). A comparison of eubacterial DGGE profiles obtained for all animal groups revealed no changes that could be attributed to a particular diet (Figure S1). However, a paired comparison of DGGE profiles supported the determination of changes resulting from restricted intake of dietary Ca or inulin intake (Figure 2). Restricted Ca intake influenced the structure of the predominant caecal microbiota in 50% of the examined animals (Figure 2a). The DGGE profiles of microbiota of four animals from groups O and R were characterised by nearly 70% similarity, whereas the microbiota in the remaining four samples from groups O and R was grouped into clusters with minimum 80% overall similarity. Cluster A1 formed by group R microbiota harboured *Olsenella* and *B. animalis* (members of the phylum Actinobacteria) and *Faecalibacterium rodentium* (*Firmicutes*), but were devoid of selected *Lachnospiraceae*- and *Peptostreptococcaceae*-specific bands which were dominant in the DGGE profiles of cluster A2 bacteria detected in four O group animals. Dietary inulin stimulated bifidobacteria, especially *B. animalis* species in group fed a GFD with the recommended amount of Ca (Figure 2b). Whereas, the addition of inulin to a calcium-deficient GFD (Figure 2c) or different Ca levels in inulin-supplemented GFDs (Figure 2d) had no effect on the banding patterns of the predominant bacteria. However, it should be stresses that in 75% of animals fed GFD with inulin, *B. animalis* was present in caecal microbiota (Figure 2d).

**Figure 3:**
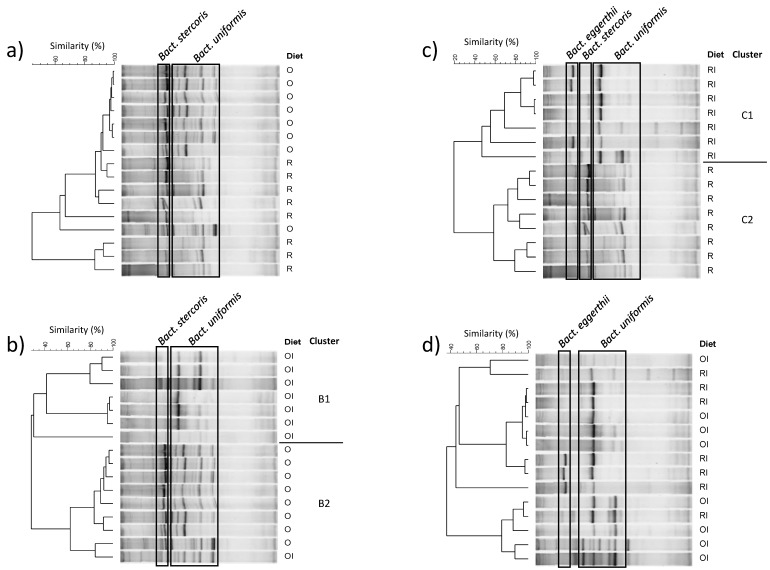
DGGE banding patterns of caecal *Bacteroides* population. Frames depict gel positions assigned to a specific bacterial taxon based on band sequencing (see Supplementary Information, Figure S1). O and R–diets with recommended and restricted calcium intake, respectively. OI and RI—diets O and R supplemented with inulin. Paired comparisons, O vs. R (**a**), O vs. OI (**b**), R vs. RI (**c**) and OI vs. RI (**d**), were performed based on profile similarities calculated using the Pearson’s product-moment correlation coefficient. Dendrograms were constructed using the UPGMA algorithm.

Similar comparisons were performed for the DGGE profiles of Bacteroides, and they revealed that inulin changed the profiles of these bacteria in groups fed GFDs with both recommended (Figure 3b) and restricted Ca levels (Figure 3c). The observed changes included the inhibition of *Bact. stercoris* (weak or absent DGGE band), a decreased representation of *Bact. uniformis* (lower number of species-specific bands; Figure 3b,c) and stimulation of *Bact. eggerthii* in group RI (Figure 3c). The limited intake of dietary Ca had no influence on the *Bacteroides* profile, and the majority of the analysed profiles revealed at least 80% similarity (Figure 3a). In the presence of inulin, different levels of dietary Ca had no effect on DGGE patterns, and most profiles had at least 80% similarity. A comparative analysis of all DGGE profiles of *Bacteroides* (Figure S2), *Lactobacillus* (Figure S3) and *C. leptum* group bacteria (Figure S4) and a paired comparison of the two latter bacterial groups (data not shown) did not reveal any effects that could be attributed to any of the tested factors.

**Figure 4:**
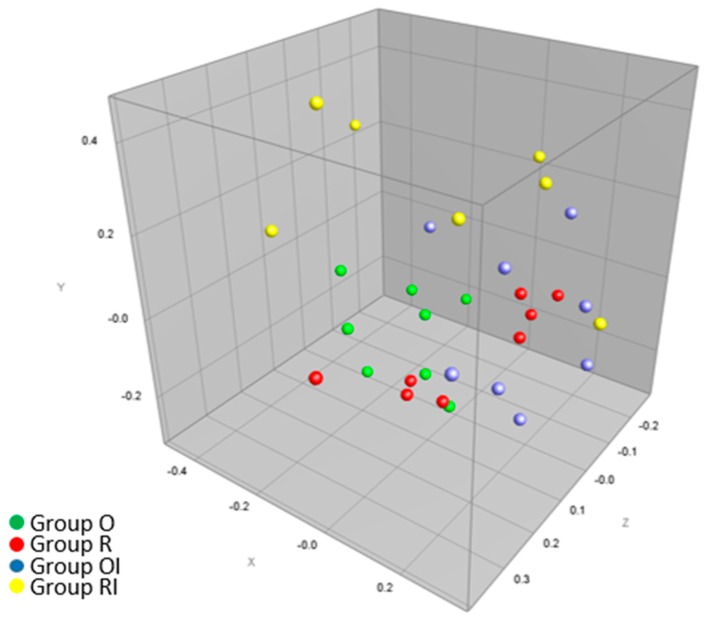
A three-dimensional MDS plot presenting the average similarities between the caecal microbiota of rats fed diets with the recommended (O, green dots) and decreased calcium levels (R, red dots) and supplemented with inulin (OI (blue dots) and RI (yellow dots), respectively). A multidimensional scaling analysis was preformed based on the similarity matrix generated during the composite data analysis of DGGE profiles of eubacteria, *Bacteroides*, *Lactobacillus* and *C. leptum* group bacteria.

A composite data set analysis was performed for the DGGE profiles of eubacteria, *Bacteroides*, *C. leptum* group and *Lactobacillus*. Multidimensional scaling of average profile similarities in the composite data set analysis revealed that caecal microbiota in the group O fed a GFD with the recommended amounts of Ca was most uniform and that supplementation of diet O with inulin (OI) scattered the samples along the y axis. A calcium-deficient GFD differentially impacted microbiota and divided the group into two subgroups (Figure 4), whereas the RI diet resulted in the most scattered distribution of samples along the x axis.

### 3.6. Effect of a Gluten-Free Diet Enriched with Inulin on Intestinal Absorption and Retention of Calcium, Magnesium and Phosphorus

The Ca supply x inulin interaction significantly influenced (*p* < 0.001) daily Ca intake (Table 5). As expected, in groups fed GFDs with reduced dietary Ca levels (R and RI), daily Ca intake was approximately 60% lower than in groups O and OI fed diets with the recommended Ca content. Two-way ANOVA revealed that faecal Ca excretion was influenced by both dietary Ca content and the presence of inulin in the experimental diets. Calcium excretion with faeces, expressed in mg/day, was significantly (*p* < 0.001) lower when dietary Ca levels were low, and it was also significantly (*p* = 0.0232) reduced by inulin intake. Consequently, relative Ca absorption (%) was significantly (*p* < 0.05) higher in groups fed a GFDs with inulin. According to statistical analysis, daily urinary Ca excretion (mg/day) was also affected by both dietary factors. Insufficient dietary Ca intake significantly decreased (*p* < 0.001) Ca excretion with urine, whereas inulin intake increased Ca urinary excretion, in particular in the group fed a GF diet with the recommended Ca level. Relative Ca retention (%) was significantly (*p* < 0.001) higher in groups fed calcium-deficient GFDs, regardless of the presence or absence of inulin. Daily magnesium (Mg) intake from GFDs was not affected by the analysed experimental variables, whereas Mg excretion with faeces and Mg absorption were significantly affected (Table 5). Faecal Mg excretion decreased significantly in response to low levels of dietary Ca as well as inulin intake (*p* < 0.001 and *p* = 0.0173, respectively), which increased relative absorption. Inulin increased urinary Mg excretion, but it did not influence the relative retention of Mg (%) which was calcium-dependant. The phosphorus balance was associated exclusively with the amount of Ca provided by the experimental GFDs, which significantly reduced faecal excretion and increased absorption in groups fed calcium-deficient diets (R and RI).

### 3.7. Effect of a Gluten-Free Diet Enriched with Inulin on Intestinal Absorption and Retention of Calcium, Magnesium and Phosphorus

Plasma Ca concentration was characterised by non-significant variations (2.337 to 2.545 mM/L), but an increasing trend was noted in groups fed inulin-supplemented GF diets (*p* = 0.0601), in particular when dietary Ca supply was restricted (Table 6). Phosphorus concentration was significantly (*p* = 0.0026) affected by the Ca × I interaction. Insufficient dietary Ca intake and inulin supplementation led to a significant (*p* < 0.001 and *p* = 0.0063) increase in plasma *P* concentration. Consequently, the Ca to phosphorus ratio decreased significantly (*p* < 0.001) in groups fed calcium-deficient GFDs.

## 4. Discussion

In the present study, all experimental GFDs had similar energy value, thus no effect on rats body weight gain was observed. Inulin in GFDs increased the weight of the small intestine and its acidification that suggested that inulin could be metabolised to a certain extent by aerotolerant small intestinal bacteria. Recently, van den Bogert et al. [36,37] demonstrated that small-intestinal streptococci *S. mitis*, *S. bovis* and *S. salivarius* differed considerably in their carbohydrate metabolism, whereas Veillonella species utilised lactic acid produced by carbohydrate-fermenting streptococci, which contributed to the synthesis of acetic and propionic acids, and led to environment acidification. 

Similarly to other prebiotics, inulin affects mainly caecal parameters by influencing resident microbiota, in particular anaerobic species [38]. In the present study, the pH of a large intestinal digesta decreased whereas the weight of caecal and colonic tissues as well as their digesta increased considerably in animals fed GFDs with inulin. The observed inulin-mediated increase in the weight of tissue of the large intestinal segments could be attributed to fructans’ ability to stimulate bacterial growth and proliferation, however in the present study, the DM content of the digesta did not increase. This indicates that apart from bacterial counts, bacterial activity considerably influenced intestinal parameters. In view of the above, the increase in the weight of caecal and colonic tissues could be partially attributed to inulin fermentation products, mainly SCFAs. Butyric acid and, to some extent, also propionic acid, are the main energy substrates for colonocytes [39]. Butyrate stimulates the physiological proliferation of enterocytes, induce histological changes in the gut epithelium and modifications of the mucosal architecture [40]. Whereas, the noted increase in the weight of large intestinal contents could have simply resulted from the inulin-mediated bulking effect and a higher content of water in the large intestinal digesta [41]. Nevertheless, a fructan-mediated increase in the weight of caecal digesta could have adverse consequences and could cause discomfort to the host [42]. On the other hand, a considerable effect on intestinal parameters could also be exerted by the level of dietary Ca intake. Recent research has demonstrated that Ca plays a number of important roles in eukaryotes as well as in prokaryotic cells [43]. Ca^2+^ ions affect the growth, division and differentiation of prokaryotic cells, and in the absence of Ca^2+^, *E. coli* cells ceased to divide and proliferate, and they were eventually lysed and died [44]. 

The caecum is a site of intensive carbohydrate fermentation as well as proteolytic activity which is largely mediated by microbiota, where ammonia is the main metabolite. In our study, an inulin-mediated decrease in a caecal ammonia concentration was noticed that could result from luminal acidification and, consequently, inhibited protein degradation in an acidic environment. Proteases are more active at neutral or slightly alkaline pH than in acidic pH [45]. In general, a reduction in caecal ammonia concentration is a favourable change since ammonia is the most toxic form of nitrogen and a metabolic disruptor [46]. The results of this study suggest that inulin and/or its fermentation products could facilitate to the utilisation and/or elimination of ammonia. 

Changes in the concentrations of SCFAs, bacterial enzyme activities and DGGE profiles of eubacteria and *Bacteroides* affirmed an intensive fermentation of inulin by caecal microbiota. The degree of polymerisation (DP) and the solubility of fructooligosaccharides are vital criteria for SCFAs formation, which is why oligosaccharides with a low DP produced high levels of butyric acid, whereas oligosaccharides with a high DP, such as inulin, produced high levels of propionic acid [47]. In this study, the high propionate to total SCFAs ratio appears to be typical for the fermentation of long-chain inulin. On the other hand, a high concentration of propionate in rats fed inulin could be linked with an abundance of *Bacteroidetes* and several *Firmicutes* species which utilise succinate as a substrate for propionate synthesis through decarboxylation of methylmalonyl-CoA to propionyl-CoA [48]. The addition of inulin to the experimental GFD increased butyrate concentration, but only in the group fed a GFD with the recommended Ca content, which suggests that such diets could create a favourable environment for *Firmicutes*. *Firmicutes* species, including *Faecalibacterium*, *Eubacterium* and *Roseburia*, are the main butyrate-producers in the colon which are able to convert butyryl-CoA to butyrate in a single-step enzymatic reaction, the butyryl-CoA: acetate CoA-transferase pathway [49]. Surprisingly, the low concentration of butyrate in group RI could be linked with changes in the utilisation of β-hydroxybutyryl-CoA. Prokaryotic cells maintain tight control of their cytosolic Ca^2+^ by means of non-proteinaceous polyhydroxybutyrate-polyphosphate (PHB-PP) complexes or Ca^2+^ channels. In the latter case, Ca ions are extruded by Ca^2+^-translocating ATPases or electrochemical potential-driven Ca^2+^ transporters [50]. β-hydroxybutyryl-CoA is one of key elements of butyrate synthesis in both butyryl-CoA:acetate CoA-transferase and butyrate kinase pathways [51]. However, it is also a precursor for PHB synthesis that relies on poly-3-hydroxybutyrate (PHB) synthase [52]. It could be hypothesized that prebiotic fermentation increases acetyl-CoA synthesis and, consequently, acetoacetyl-CoA and β-hydroxybutyryl-CoA. This leads to a metabolic switch from butyrate production to the synthesis of PHB which acts as a universal regulator of internal ion concentrations by selectively transporting ions across membranes [53]. It is likely that a calcium-deficient GFD without inulin is not a sufficient source of carbohydrates for the production of acetyl-CoA in amounts that could be metabolically economic for the butyrate→PHB switch. Therefore, bacteria cope with low Ca levels by activating a proteinaceous system of Ca transport [50]. On the other hand, it cannot be ruled out that nearly all available butyrate was used up by the enlarged intestinal tissue and that butyrate levels in the intestinal lumen were depleted. This observation is supported by the lowest values of Ca excretion with faeces and highest Ca absorption. Further research is needed to verify the above hypotheses.

SCFAs deliver health benefits [54]. The possible connection between selected inflammatory processes and a reduction in the populations of butyrate- and propionate-producing bacteria has turned the researchers’ attention to the metabolism of propionate and butyrate. Machiels et al. [55] reported a decrease of in the counts of butyrate-producing species in ulcerative colitis. Based on above, it could be expected that the acidification of the large intestine resulting from SCFAs production could promote the growth and proliferation of beneficial microbiota and reduce the number of potentially pathogenic species. Our speculations were also supported by the results of a quantitative analysis of branched SCFAs which revealed that the addition of inulin to a GFDs suppressed putrefaction processes when the intake of dietary Ca was low.

In animals fed a GFD with the required Ca content, inulin exerted a similar influence on microbiota (stimulation of *B. animalis*) to that reported in other studies [56]. An analysis of eubacterial DGGE patterns indicates that members of the phylum Actinobacteria (*Olsenella* and *Bifidobacterium*) seem to be less susceptible to low Ca levels than *Firmicutes*, in particular the family *Lachnospiraceae*. Moreover, the MDS analysis based on DGGE profiles revealed that limited intake of dietary Ca was associated with two microbiological phenotypes, which points to individual variations in response to low Ca levels. Moreover, the combined effect of dietary factors (restricted dietary Ca intake and inulin intake) led to the most scattered distribution of samples. Therefore, it could be assumed that inulin together with low Ca levels trigger substantial changes in microbial structure due to individual differences in microbial composition. The observed in the present study changes in the structure and metabolic activity of caecal microbiota seem to indicate that the combination of low Ca intake and inulin has an undesirable effect on intestinal bacteria. The presence of *Bacteroides eggerthii*, which was recently proposed as a colitis-promoting species [57], could be of particular interest in further studies on maintaining a properly balanced commensal microbiota. 

The inulin-mediated modification of gut microbiota was also manifested by changes in the activity of bacterial enzymes in the caecum. The experimental GFDs with inulin increased the concentration of α- and β-glucosidase and β-galactosidase, whereas the activity of β-glucuronidase was suppressed in groups fed GFDs with inulin. Similar results were reported in other studies of fructooligosaccharides [42,58] which demonstrated that moderate FOS content significantly reduced the caecal activity of β-glucuronidase and increased the activity of β-galactosidase. Bile flow modulates the activity of β-glucuronidase in the large intestines of rats [59], therefore, the inulin-induced decrease in β-glucuronidase activity could be attributed to intensified intestinal peristalsis. In general, a reduction in β-glucuronidase activity is beneficial because this enzyme exerts toxic, carcinogenic, and mutagenic effects in the gastrointestinal tract [60]. Thus, the addition of inulin to the experimental GF diets seems to selectively modulate the composition of microbiota, and it potentially eliminates harmful bacteria that enhance the activity of β-glucuronidase in the caecum.

Fermentable carbohydrates, including inulin, were found to increase Ca and Mg absorption in the large intestine [21,22], but this effect was dependent on the dose, structure of fermentable carbohydrates as well as the duration of the experiment. In the present study, we hypothesised that the addition of inulin to a GFD would increase Ca absorption in the large intestine, in particular in animals with low dietary Ca intake. Inulin intake stimulated Ca and Mg absorption in the group fed a calcium-deficient GFD however, the increase in Ca and Mg absorption was not accompanied by changes in their retention. Coudry et al. [23] found that dietary inulin had a more beneficial effect on Ca absorption in shorter-term (17 days) than in long-term experiments (up to 40 days), and that inulin-mediated changes were more profound when dietary Ca was in low supply. In contrast, in another study, a 6-week prebiotic intervention (oligofructose + acacia gum) had no effect on Ca absorption or Ca retention in aged ovariectomized rats [61]. In the present study performed on growing rats, which are characterised by a higher demand for Ca and higher Ca absorption capacity, the increase in Ca and Mg absorption could be attributed to prolonged inulin fermentation in the caecum, which was manifested by changes in large intestinal parameters, mainly an increase in SCFA synthesis and acidification of caecal and colonic contents. This indicates that acidic caecal pH promotes the solubilisation of Ca and Mg. The observed increase in Ca absorption in animals fed GFD with inulin is a promising result that could generate favourable surplus Ca for bone, however further in vivo studies are required to assess the impact of a dietary application of inulin on calcium metabolism and bone mineralisation. 

## 5. Conclusions

Our results indicated that the effect of inulin on intestinal microbiota characteristics and activity, and mineral utilization in growing rats depended on the dietary Ca intake in GFDs. Generally, dietary inulin stimulated the SCFAs formation, increased the luminal acidification and decreased caecal ammonia concentration. However, unsufficient Ca intake in GFD influenced negatively the structure of the predominant caecal microbiota, while a dietary inulin stimulated bifidobacteria, in particular *B. animalis* species if recommended amount of Ca was provided in rats diet. Most benefits to mineral utilization from inulin consumption were seen in rats fed GFD of restricted Ca amount where it increased the relative Ca absorption. Obtained results allow to conclude that the administration of inulin to a GFDs could be a promising dietary strategy for beneficial modulation of intestinal ecosystem and by that for the improvement the Ca absorption. 

## Figures and Tables

**Table 1:** Experimental gluten-free diets.

	O	R	OI	RI
Bread ingredients as diet compounds (%)				
Corn starch	48.4	49.8	40.4	41.8
Potato starch	11.7	11.7	11.7	11.7
Pectin	3.0	3.0	3.0	3.0
Sugar	3.7	3.7	3.7	3.7
Salt	1.0	1.0	1.0	1.0
Sunflower oil	1.7	1.7	1.7	1.7
Yeast	3.7	3.7	3.7	3.7
CAS *	12.0	12.0	12.0	12.0
CIT ^&^	2.0	0.6	2.0	0.6
Inulin ^#^	0.0	0.0	8.0	8.0
Other diet compounds (%)				
dl-Methionine	0.3	0.3	0.3	0.3
Soya oil	8.0	8.0	8.0	8.0
Ca-free mineral mix ^†^	3.5	3.5	3.5	3.5
Vitamin mix ^§^	1.0	1.0	1.0	1.0
Energy density (kcal/g)	4.01	4.00	3.82	3.80

O—GFD of recommended Ca content; OI—GFD of recommended Ca content with inulin; R—GFD of reduced Ca content; RI—GFD of reduced Ca content with inulin, * Calcium caseinate (contained: 92.8% protein, 2.06% fat, 4.01% ash, 5.12% moisture), ^&^ Calcium citrate (E 333(iii); contained 21.98 ppm of Ca), ^#^ Frutafit HD; contained 99.5% carbohydrates (≥90% inulin, ≤10% fructose, glucose, sacchcrose), ^†^ Mineral mix AIN-93G-MX without Ca [29], ^§^ Vitamin mix AIN-93G-VM [29].

**Table 2:** Daily food intake, body weight (BW) gain and gastrointestinal tract parameters in rats fed experimental GF diets *.

	Diets				Ca Effect	Inulin Effect	Ca × I
	O	R	OI	RI			
Daily food intake (g/animal)	13.90 ± 1.22	13.15 ± 1.56	13.83 ± 0.54	13.26 ± 0.95	0.0688	0.7049	0.9054
Daily BW gain (g/animal)	4.55 ± 0.45	4.61 ± 0.53	4.42 ± 0.30	4.38 ± 0.34	0.9477	0.2312	0.7594
Small intestinal parameters							
Weight (g/100 g BW)	3.10 ± 0.13	2.86 ± 0.19	3.26 ± 0.32	3.19 ± 0.34	0.0983	0.0139	0.3633
pH of small intestinal digesta	7.03 ± 0.22	7.02 ± 0.18	6.86 ± 0.20	6.67 ± 0.24	0.1991	0.0015	0.2282
Caecum parameters							
Weight of tissue (g/100 g BW)	0.320 ± 0.03 ^c^	0.332 ± 0.03 ^c^	0.436 ± 0.04 ^b^	0.587 ± 0.08 ^a^	<0.001	<0.001	<0.001
Weight of digesta (g/100 g BW)	1.124 ± 0.198 ^b^	1.112 ± 0.202 ^b^	1.441 ± 0.240 ^b^	3.110 ± 0.694 ^a^	<0.001	<0.001	<0.001
DM content of digesta (%)	16.79 ± 1.34	15.65 ± 1.42	15.50 ± 1.33	14.79 ± 1.40	0.0671	0.0346	0.6537
Ammonia (mg/g digesta)	0.191 ± 0.026	0.183 ± 0.018	0.141 ± 0.016	0.154 ± 0.022	0.7069	<0.001	0.1704
pH of digesta	6.79 ± 0.13	6.53 ± 0.18	6.18 ± 0.24	5.83 ± 0.39	0.0019	<0.001	0.5949
Colonic parameters							
Weight of tissue (g/100 g BW)	0.531 ± 0.072 ^a,b^	0.467 ± 0.042 ^b^	0.535 ± 0.055 ^a,b^	0.621 ± 0.094 ^a^	0.6517	0.0028	0.0044
Weight of digesta (g/100 g BW)	0.519 ± 0.108 ^b^	0.389 ± 0.153 ^c^	0.570 ± 0.095 ^a,b^	0.658 ± 0.078 ^a^	0.5933	<0.001	0.0104
pH of digesta	6.71 ± 0.186	6.11 ± 0.178	6.11 ± 0.168	5.66 ± 0.132	<0.001	<0.001	0.2046

Values are expressed as means ± standard deviation. * O, group fed GFD with the recommended calcium content; R, group fed GFD with restricted calcium content; OI, group fed GFD with the recommended calcium content and inulin; RI, group fed GFD with restricted calcium content and inulin. BW, body weight. ^a,b,c^ Mean values in rows with different superscript letters are significantly different (*p* < 0.05). The differences between groups O, R, OI and RI groups are indicated with superscripts only when Ca × I interactions were statistically significant (*p* < 0.05).

**Table 3:** The concentration of short chain fatty acids (SCFAs), putrefactive SCFAs (PSCFAs) and their profiles in the caecal digesta of rats fed experimental GFDs *.

	Diets				Ca Effect	Inulin Effect	Ca × I
	O	R	OI	RI			
SCFAs (μM/g digesta)							
Acetic	55.17 ± 7.19 ^b^	53.22 ± 9.46 ^b^	58.49 ± 8.56 ^a^	35.14 ± 8.94 ^c^	<0.001	0.0216	0.0015
Propionic	16.30 ± 1.39 ^c^	28.51 ± 6.15 ^b^	39.91 ± 7.65 ^a^	27.57 ± 7.48 ^b^	0.9768	<0.001	<0.001
Iso-butyric	0.41 ± 0.22	0.17 ± 0.07	0.29 ± 0.13	0.05 ± 0.03	<0.001	0.0187	0.9336
Butyric	6.09 ± 2.02 ^b^	5.68 ± 0.72 ^b^	8.92 ± 2.79 ^a^	2.31 ± 1.64 ^c^	<0.001	0.6953	<0.001
Iso-valeric	0.70 ± 0.21	0.44 ± 0.17	0.25 ± 0.13	0.14 ± 0.06	0.0016	<0.001	0.1738
Valeric	1.28 ± 0.24	0.67 ± 0.14	0.64 ± 0.18	0.05 ± 0.02	<0.001	<0.001	0.5737
PSCFAs	2.39 ± 0.50	1.28 ± 0.21	1.19 ± 0.29	0.23 ± 0.08	<0.001	<0.001	0.4801
Total SCFAs	79.95 ± 9.43 ^b^	88.69 ± 13.98 ^b^	108.50 ± 13.95 ^a^	65.31 ± 17.43 ^c^	0.0017	0.6097	<0.001
C2:C3:C4 profile (%)							
C2	69 ^a^	60 ^b^	54 ^c^	54 ^c^	<0.001	<0.001	<0.001
C3	21 ^c^	32 ^b^	37 ^b^	42 ^a^	<0.001	<0.001	0.0070
C4	8 ^a^	6 ^b^	8 ^a^	4 ^c^	<0.001	0.1097	0.0086

Values are expressed as means ± standard deviation. * O, group fed GFD with the recommended calcium content; R, group fed GFD with restricted calcium content; OI, group fed GFD with the recommended calcium content and inulin; RI, group fed GFD with restricted calcium content and inulin. BW, body weight. ^a,b,c^ Mean values in rows with different superscript letters are significantly different (*p* < 0.05). The differences between groups O, R, OI and RI groups are indicated with superscripts only when Ca × I interactions were statistically significant (*p* < 0.05).

**Table 4:** The activity of bacterial enzymes in the caecal digesta of rats fed the experimental GFDs *.

	Diets				Ca Effect	Inulin Effect	Ca × I
	O	R	OI	RI			
α-Glucosidase (μmol/h/g)	13.86 ± 7.28	31.80 ± 13.76	29.74 ± 10.55	40.72 ± 10.99	<0.001	0.0032	0.3737
β-Glucosidase (μmol/h/g)	4.94 ± 1.49 ^b^	7.53 ± 1.30 ^a^	6.90 ± 1.74 ^a^	3.82 ± 0.71 ^b^	0.6205	0.0803	<0.001
α-Galactosidase (μmol/h/g)	7.86 ± 1.71	4.48 ± 0.92	8.22 ± 3.58	7.84 ± 4.92	0.1066	0.1096	0.1939
β-Galactosidase (μmol/h/g)	27.37 ± 8.85	44.19 ± 10.08	48.99 ± 19.22	56.56 ± 19.59	0.0319	0.0039	0.3995
β-Glucuronidase (μmol/h/g)	18.82 ± 6.14 ^a^	4.77 ± 1.98 ^b^	5.67 ± 2.71 ^b^	1.85 ± 0.98 ^c^	<0.001	<0.001	<0.001

Values are expressed as means ± standard deviation. * O, group fed GFD with the recommended calcium content; R, group fed GFD with restricted calcium content; OI, group fed GFD with the recommended calcium content and inulin; RI, group fed GFD with restricted calcium content and inulin. BW, body weight. ^a,b,c^ Mean values in rows with different superscript letters are significantly different (*p* < 0.05). The differences between groups O, R, OI and RI groups are indicated with superscripts only when Ca × I interactions were statistically significant (*p* < 0.05).

**Table 5:** Intestinal absorption and retention of calcium, magnesium and phosphorus in rats fed experimental GF diets *.

	Diets				Ca Effect	Inulin Effect	Ca × I
	O	R	OI	RI			
Calcium							
Intake (mg/day)	86.21 ± 7.87 ^a^	35.64 ± 4.24 ^b^	80.46 ± 5.37 ^a^	33.95 ± 2.44 ^b^	<0.001	0.0024	0.0220
Faecal excretion (mg/day)	22.68 ± 4.08	2.01 ± 0.43	20.05 ± 4.43	0.84 ± 0.32	<0.001	0.0232	0.2026
Absorption (%) ^†^	73.28 ± 3.13	94.36 ± 0.80	74.60 ± 4.86	97.51 ± 0.92	<0.001	0.0414	0.3879
Urinary excretion (mg/day)	6.92 ± 1.98	0.54 ± 0.15	9.44 ± 2.98	0.52 ± 0.18	<0.001	0.0179	0.0673
Retention (%) ^‡^	65.63 ± 2.23	93.05 ± 1.84	62.62 ± 6.18	94.18 ± 3.80	<0.001	0.5916	0.0753
Magnesium							
Intake (mg/day)	6.35 ± 0.56	6.30 ± 0.75	6.46 ± 0.25	6.21 ± 0.43	0.1695	0.9289	0.2754
Faecal excretion (mg/day)	1.72 ± 0.37	0.42 ± 0.07	1.48 ± 0.19	0.32 ± 0.06	<0.001	0.0173	0.3777
Absorption (%) ^†^	72.94 ± 5.06	93.28 ± 0.99	76.54 ± 1.50	95.21 ± 1.26	<0.001	0.0078	0.3920
Urinary excretion (mg/day)	3.03 ± 0.24	3.11 ± 0.41	3.25 ± 0.28	3.27 ± 0.47	0.4112	0.0321	0.2432
Retention (%) ^‡^	28.54 ± 8.57	43.84 ± 3.30	28.51 ± 4.04	43.68 ± 5.07	<0.001	0.4611	0.7504
Phosphorus							
Intake (mg/day)	35.26 ± 3.10	34.74 ± 4.13	35.44 ± 2.42	32.79 ± 2.35	0.1515	0.4407	0.3269
Faecal excretion (mg/day)	13.19 ± 1.54	4.68 ± 0.73	12.62 ± 1.83	4.57 ± 0.24	<0.001	0.0517	0.9248
Absorption (%) ^†^	62.66 ± 2.79	86.42 ± 2.25	63.23 ± 1.05	88.90 ± 3.48	<0.001	0.0994	0.2963
Urinary excretion (mg/day)	0.37 ± 0.07	9.37 ± 1.39	0.37 ± 0.15	8.91 ± 0.74	<0.001	0.2644	0.2606
Retention (%) ^‡^	61.60 ± 2.79	60.21 ± 3.01	63.62 ± 4.13	62.30 ± 8.00	0.0927	0.0314	0.7850

* Values are expressed as means ± standard deviation. O, group fed a GF diet with the recommended calcium content; R, group fed a GF diet with restricted calcium content; OI, group fed a GF diet with the recommended calcium content and inulin; RI, group fed a GF diet with restricted calcium content and inulin. ^†^ Absorption: [(Intake—Faecal excretion)/Intake] × 100. ^‡^ Retention: [(Intake—Faecal excretion-Urinary excretion)/Intake] × 100. ^a,b,c^ Mean values in rows with different superscript letters are significantly different (*p* < 0.05). The differences between groups O, R, OI and RI are indicated with superscripts only when Ca × I interactions were statistically significant (*p* < 0.05).

**Table 6:** Plasma calcium and phosphorus concentration in rats fed experimental GFDs *.

	Diets				Ca Effect	Inulin Effect	Ca × I
	O	R	OI	RI			
Calcium (mM/L)	2.522 ± 0.084	2.337 ± 0.059	2.545 ± 0.204	2.537 ± 0.238	0.1083	0.0662	0.1393
Phosphorus (mM/L)	1.861 ± 0.247 ^b^	2.202 ± 0.166 ^a^	1.835 ± 0.211 ^b^	2.670 ± 0.215 ^a^	<0.001	0.0063	0.0026
Ca:P	1.4	1.1	1.4	1.0	<0.001	0.3116	0.1177

* Values are expressed as means ± standard deviation. O, group fed a GF diet with the recommended calcium content; R, group fed a GF diet with restricted calcium content; OI, group fed a GF diet with the recommended calcium content and inulin; RI, group fed a GF diet with restricted calcium content and inulin. ^a,b,c^ Mean values in rows with different superscript letters are significantly different (*p* < 0.05). The differences between groups O, R, OI and RI are indicated with superscripts only when Ca × I interactions were statistically significant (*p* < 0.05).

## Supplementary Materials

The following are available online at www.mdpi.com/2072-6643/9/7/702/s1, Figure S1: DGGE profiles of rats’ caecal bacteria obtained with universal primers. Rats were fed diet with optimal (O) or restricted (R) calcium supply supplemented with inulin (diet OI and RI, respectively). Arrows indicate band taken for identification which results are shown in a table below. Figure S2. DGGE profiles of rats’ caecal *Bacteroides* population. Rats were fed diet with optimal (O) or restricted (R) calcium supply supplemented with inulin (diet OI and RI, respectively). Arrows indicate band taken for identification which results are shown in a table below. Figure S3. DGGE profiles of rats’ caecal *Lactobacillus* population. Rats were fed diet with optimal (O) or restricted (R) calcium supply supplemented with inulin (diet OI and RI, respectively). Arrows indicate band taken for identification which results are shown in a table below. Figure S4. DGGE profiles of rats’ caecal *C. leptup* group (clostridial cluster IV). Rats were fed diet with optimal (O) or restricted (R) calcium supply supplemented with inulin (diet OI and RI, respectively). Table S1: Nutritional composition of experimental gluten-free diets, Table S2: Primers and amplification conditions used for qualitative (PCR-DGGE) analysis of caecal microbiota, Table S3: Denaturing gradient applied for separation of PCR products in DGGE technique; Table S4: Primers used for real-time PCR analysis.
