# Supplementary material for: Administration of Inulin-Supplemented Gluten-Free Diet Modified Calcium Absorption and Caecal Microbiota in Rats in a Calcium-Dependent Manner
Source: Nutrients. 2017 Jul 6;9(7):702. doi: 10.3390/nu9070702 (PMC5537817; doi:10.3390/nu9070702)
Supplement: Supplementary file 1 [file nutrients-09-00702-s001.zip › Supplementary Information Materials and Methods.pdf]

Supplementary Information

S1. Materials and Methods

S1.1. Nutritional characteristics of gluten-free diets

The dry matter, proteins, ash, and total starch (Table S1) of experimental GFDs were determined according to the methods 935.36, 950.36, 930.22, and polarization method 945.37, respectively [1]. The fat content was calculated by subtracting the sum of total solids (100 – proteins – ash - total starch - inulin; expressed in g/100g). Based on literature date, it was assumed that the calorific value of inulin is 1.5 kcal/g [2]. Mineral concentrations in diets (Table S1) was measured by flame (air – acetylene burner) atomic absorption spectrometry method (AAS) using an atomic absorption spectrophotometer (iCE 3000 SERIES - THERMO - England) equipped with an autosampler and the appropriate cathode lamp operating at the resonance line of the analysed bioelements (Ca: 422.7 nm; Mg: 285.2 nm). Before analysis, the samples were wet-digested with a mixture (9 : 1; v/v) of concentrated nitric acid (65 % HNO3; Merck, Darmstadt, Germany) and hydrochloric acid (30 % HCl; Merck) using a microwave system (Multiwave, Anton Paar GmbH, Graz, Austria). Calcium concentration was validated by adding a solution of lanthanum (III) chloride hydrate (LaCl3 x 7H2O; Merck, Germany) to all samples in sufficient amounts to obtain 0.5% concentration of La3+. Phosphorus content was determined by the colorimetric molybdate method with hydroquinone (POCH S. A. Gliwice, Poland) and sodium (IV) sulphate (POCH S. A. Gliwice, Poland). Absorbance was measured using the VIS 6000 Spectrophotometer (KRÜSS– OPTRONIC, Germany) at $\lambda = 610$ nm. The concentrations of Ca, Mg and P were automatically read from a calibration curve (Ca: range 0.5 – 4.0 μg/mL; Mg: range 0.05 – 0.8 μg/mL; P: range 0.4 –2.0 μg/mL) prepared with the AAS standard solution of Ca, Mg and P, respectively (J.T.Baker®, Holland). The analyses were repeated (N = 8) for analytical quality control.

Table S1. Nutritional composition of experimental gluten-free diets

	O	R	OI	RI
Content of macronutrients [g/100g]				
Dry matter	9.44 ± 0.05	9.32 ± 0.04	9.26 ± 0.14	9.01 ± 0.07
Proteins*	13.97 ± 0.62	13.94 ± 0.71	13.68 ± 0.39	14.09 ± 0.44
Starch	65.06 ± 0.39	66.36 ± 0.43	57.80 ± 1.20	59.09 ± 0.68
Ash	2.10 ± 0.11	1.57 ± 0.08	1.94 ± 0.10	1.51 ± 0.04
Content of selected minerals [mg/g]				
Calcium	6.44 ± 0.03	2.71 ± 0.02	5.58 ± 0.01	2.56 ± 0.01
Magnesium	0.46 ± 0.003	0.48 ±0.001	0.47 ± 0.001	0.46 ±0.001
Phosphorus	2.54 ± 0.03	2.64 ± 0.02	2.51 ± 0.01	2.47 ± 0.00

* N x factor 6.25

S1.2. *Microbiota characteristics with PCR-DGGE*

Table S2. Primers and amplification conditions used for qualitative (PCR-DGGE) analysis of caecal microbiota.

Name	Sequence (5′ Ÿ 3′)	PCR product (bp)	$MgCl_2$ (mM)	T_a (°C)	Target	Reference
1401-r<br>968-f-GC	cggtgtgtacaagaccc<br>GC-aacgcgaagaacctta	486	5.0	58	Eubakteria	[3]
Bfrag-F<br>Bfrag-R-GC	aacgctagctacaggctt<br>GC-caatcggagttcttcgtg	388	1.5	56	*Bacteroides*	[4]
CLept-F<br>CLept-R-GC	gcacaagcagtggagt<br>GC-cttcctccgttttgtcaa	239	2.0	53	*Clostridium leptum* group	[5]
Lac1F<br>Lac2 GC R	agcagtagggaatcttcca<br>GC-attycaccgctacacatg	347	5.0	57	*Lactobacillus*	[6]
GC clamp	cgcccggggcgcgccggcggcggcccgggggcaccggggg		-	-		[7]

Table S3. Denaturing gradient applied for separation of PCR products in DGGE technique.

Bacterial group	Gradient (%)	Electrophoretical conditions
Eubakteria	25-65	200V, 10 min.; 85V, 18 h
Bacteroides	25-55	200, 10 min.; 85V, 16 h
Clostridium leptum group	25-37.5	85V, 10 min; 200V, 4 h
Lactobacillus	40-55	200V, 10 min.; 85V, 20 h

S1.3. *Quantification of caecal microbiota by real-time PCR*

Table S4. Primers used for real-time PCR analysis.

Target	Primer	Sequence (5′ Ÿ 3′)	T_a (°C)	Reference
Bacteroides–Prevotella–Porphyromonas group	BPP-F<br>BPP-R	ggtgtcggcttaagtgccat<br>cgga(c/t)gtaagggccgtgc	56	[8]
Bifidobacterium	BIF-F<br>BIF-R	tcg cgtc(c/t)ggtgtgaaag<br>ccacatccagc(a/g)tccac	58	[8]
Clostridium leptum group	sg-Clept-F<br>sg-Clept-R3	gcacaagcagtggagt<br>cttcctccgttttgtcaa	53	[5]
Enterococcus	ECC-F<br>ECC-R	cccttattgttagttgccatcatt<br>actcgttgtacttcccattgt	61	[8]
Lactobacillus	Lac1F<br>Lab667	agcagtagggaatcttcca<br>caccgctacacatggag	58	[7]
Total bacteria	UniF<br>UniR	gtgstgcayggyygtcgtca<br>acgtcrtccmcnccttcctc	60	[9]

References

1. Official Methods of Analysis of AOAC International, 2005, 18th Edition, 2005, Horwitz W., Latimer Jr. G.W. (eds), Gaithersburg, Maryland, USA
2. Roberfroid, M.B. Caloric value of inulin and oligofructose *J Nutr* **1999**, *129*, 1436S–1437S.
3. Satokari, R.M.; Vaughan, E.E.; Favier, C.F.; Dore, J.; Edwards, C.; de Vos, W.M. Diversity of *Bifidobacterium* and *Lactobacillus* spp. in breast-fed and formula-fed infants as assessed by 16S rDNA sequence differences. *Microb Ecol Health Dis* **2002**, *14*, 97-105.
4. Matsuki, T.; Watanabe, K.; Fujimoto, J.; Takada, T.; Tanaka, R.; Use of 16S rRNA gene-targeted group-specific primers for real-time PCR analysis of predominant bacteria in human feces. *Appl Environ Microbiol* **2004**, *70*, 7220-7228.
5. Shen, J.; Zhang, B.; Wei, G.; Pang, X.; Wei, H.; Li, M.; Zhang, Z.; Jia, W.; Zhao, L. Molecular profiling of the *Clostridium leptum* subgroup in human fecal microflora by PCR-Denaturing Gradient Gel Electrophoresis and clone library analysis. *Appl Environ Microbiol* **2006**, *72*, 5232–5238.
6. Walter, J.; Hertel, C.; Tannock, G.W.; Lis, C.M.; Munro, K.; Hammes, W.P. Detection of *Lactobacillus*, *Pediococcus*, *Leuconostoc*, and *Weissella* species in human feces by using group-specific PCR primers and denaturing gradient gel electrophoresis. *Appl Environ Microbiol* **2001**, 67, 2578-85.
7. Heilig, H.G.J.; Zoetendal, E.G.; Vaughan, E.E.; Marteau, P.; Akkermans, A.D.L.; de Vos, W.M. Molecular diversity of *Lactobacillus* spp. and other lactic acid bacteria in the human intestine as determined by specific amplification of 16S ribosomal DNA. *Appl Environ Microbiol* **2002**, *68*, 114–123.
8. Rinttila, T.; Kassinen, A.; Malinen, E.; Krogius, L.; Palva, A. Development of an extensive set of 16S rDNA-targeted primers for quantification of pathogenic and indigenous bacteria in faecal samples by real-time PCR. *J Appl Microbiol* **2004**, *97*, 1166–1177.
9. Fuller, Z.; Louis, P.; Mihajlovski, A.; Rungapamestry, V.; Ratcliffe, B.; Duncan, A. J. Influence of cabbage processing methods and prebiotic manipulation of colonic microflora on glucosinolate breakdown in man. *Brit J Nutr* **2007**, *98*, 364-72.
