# Supplementary material for: Administration of Inulin-Supplemented Gluten-Free Diet Modified Calcium Absorption and Caecal Microbiota in Rats in a Calcium-Dependent Manner
Source: Nutrients. 2017 Jul 6;9(7):702. doi: 10.3390/nu9070702 (PMC5537817; doi:10.3390/nu9070702)
Supplement: Supplementary file 1 [file nutrients-09-00702-s001.zip › Supplementary Information Results Figures.pdf]

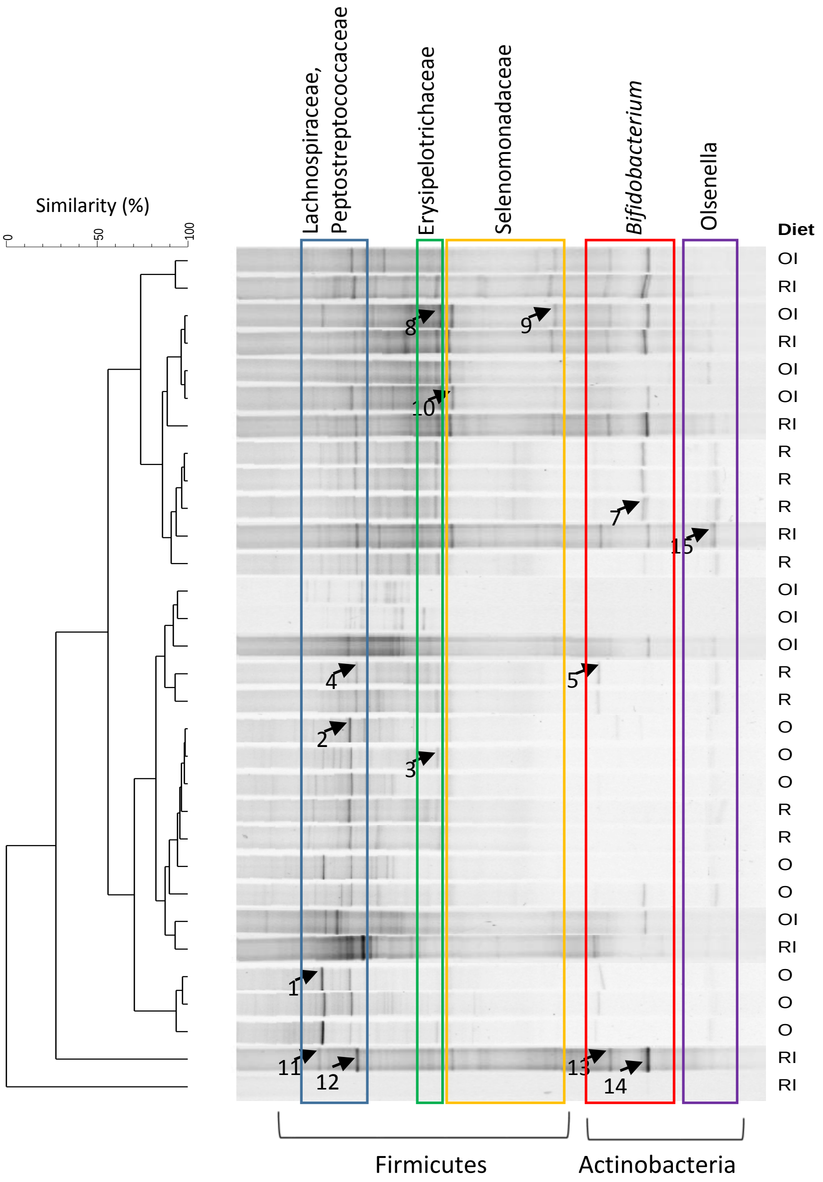

Band no	Band identification [phylum, family]
1	*Eisenbergiella massiliensis* [Firmicutes, Lachnospiraceae]
2	*Romboutsia ilealis* [Firmicutes, Peptostreptococcaceae]
3, 8	*Faecalibaculum rodentium* [Firmicutes, Erysipelotrichaceae]
4, 11, 12	*Clostridium clostridioforme* [Firmicutes, Lachnospiraceae]
5	*Bifidobacterium pseudolongum* [Actinobacteria, Bifidobacteriaceae]
7, 13, 14	*Bifidobacterium animalis* [Actinobacteria, Bifidobacteriaceae]
9	*Anaerovibrio* sp. [Firmicutes, Selenomonadaceae]
10	*Anaerovibrio lipolyticus* [Firmicutes, Selenomonadaceae]
15	*Olsenella* sp. [Actinobacteria, Atopobiaceae]

Figure S1. DGGE profiles of rats' caecal bacteria obtained with universal primers. Rats were fed diet with optimal (O) or restricted (R) calcium supply supplemented with inulin (diet OI and RI, respectively). Arrows indicate band taken for identification which resuts are shown in a table below.

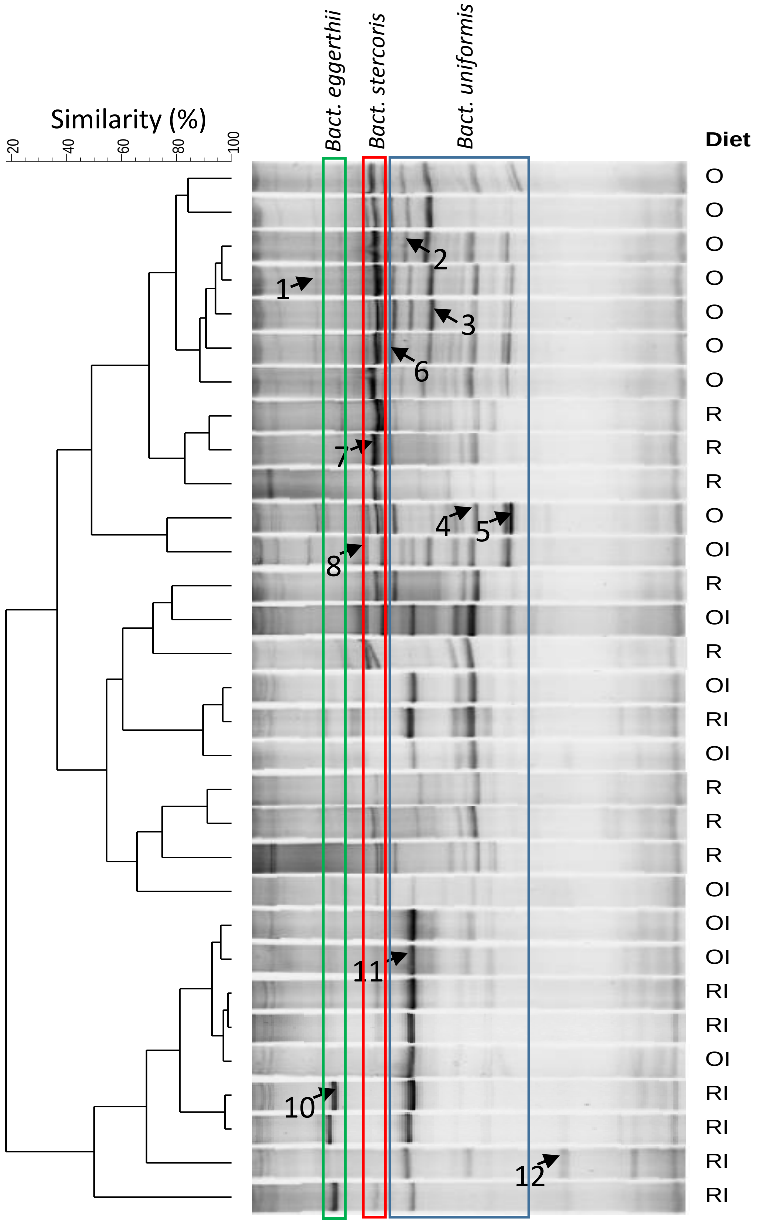

Band no	Band identification
1, 7, 12	*Bacteroides stercoris*
2	*Bacteroides acidifaciens*
3, 4, 5, 6, 8, 11	*Bacteroides uniformis*
10	*Bacteroides eggerthii*

Figure S2. DGGE profiles of rats' caecal *Bacteroides* population. Rats were fed diet with optimal (O) or restricted (R) calcium supply supplemented with inulin (diet OI and RI, respectively). Arrows indicate band taken for identification which resuts are shown in a table below.

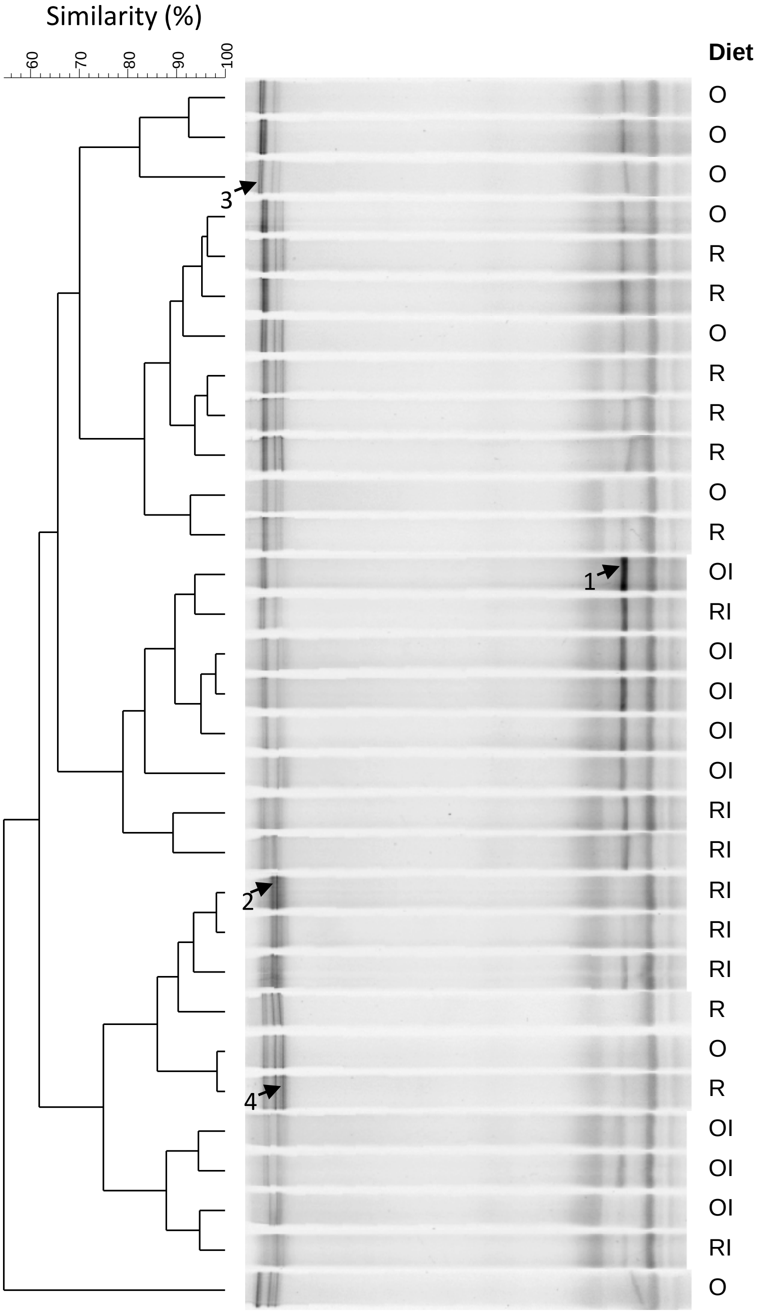

Band no	Band identification
1, 3	*Lactobacillus reuteri*
2	*Pediococcus acidilactici*
4	*Lactobacillus murinus*

Figure S3. DGGE profiles of rats' caecal *Lactobacillus* population. Rats were fed diet with optimal (O) or restricted (R) calcium supply supplemented with inulin (diet OI and RI, respectively). Arrows indicate band taken for identification which resuts are shown in a table below.

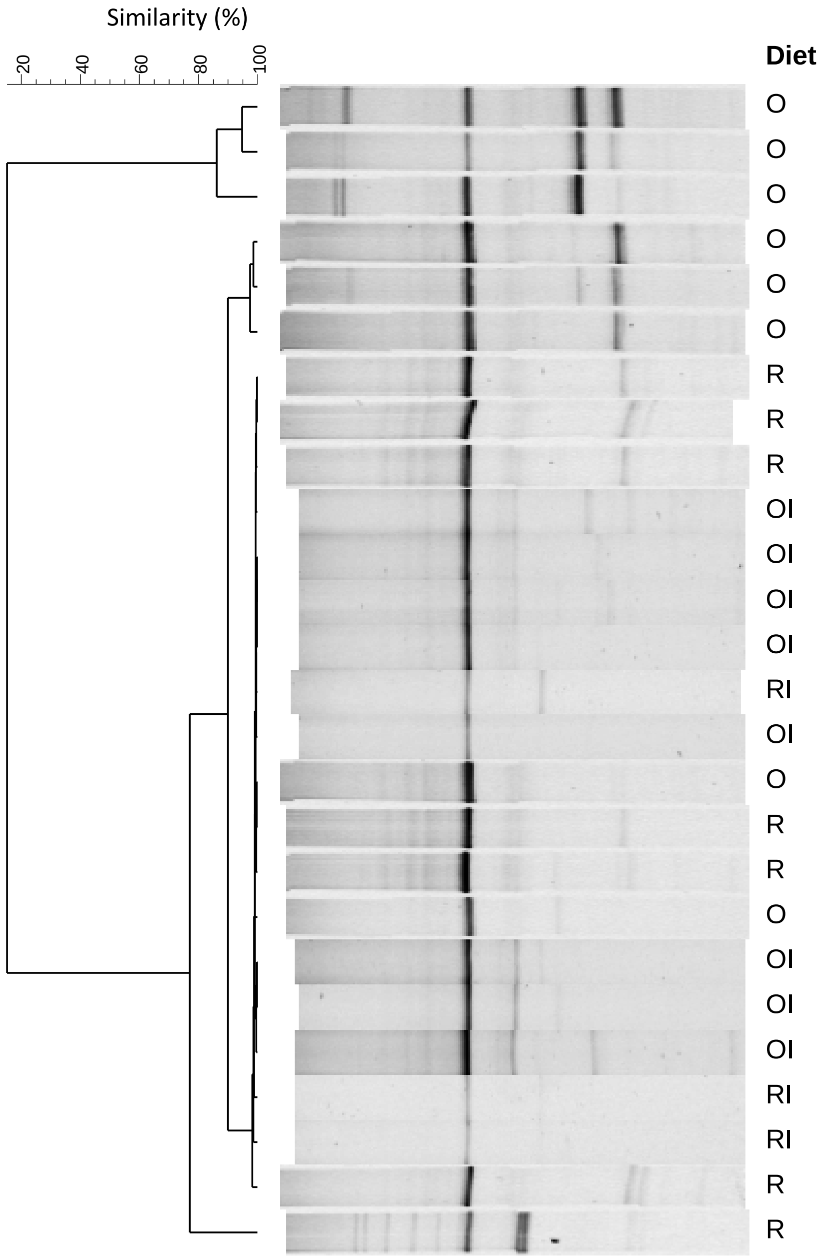

Figure S4. DGGE profiles of rats' caecal *C. leptup* group (clostridial cluster IV). Rats were fed diet with optimal (O) or restricted (R) calcium supply supplemented with inulin (diet OI and RI, respectively).
